# gRDF: An Efficient Compressor with Reduced Structural Regularities That Utilizes gRePair

**DOI:** 10.3390/s22072545

**Published:** 2022-03-26

**Authors:** Tangina Sultana, Young-Koo Lee

**Affiliations:** Department of Computer Science and Engineering, Kyung Hee University, Global Campus, Yongin-si 17104, Korea; tangina@khu.ac.kr

**Keywords:** compression, graph, gRePair, *k*^2^-trees, RDF

## Abstract

The explosive volume of semantic data published in the Resource Description Framework (RDF) data model demands efficient management and compression with better compression ratio and runtime. Although extensive work has been carried out for compressing the RDF datasets, they do not perform well in all dimensions. However, these compressors rarely exploit the graph patterns and structural regularities of real-world datasets. Moreover, there are a variety of existing approaches that reduce the size of a graph by using a grammar-based graph compression algorithm. In this study, we introduce a novel approach named gRDF (graph repair for RDF) that uses gRePair, one of the most efficient grammar-based graph compression schemes, to compress the RDF dataset. In addition to that, we have improved the performance of HDT (header-dictionary-triple), an efficient approach for compressing the RDF datasets based on structural properties, by introducing modified HDT (M-HDT). It can detect the frequent graph pattern by employing the data-structure-oriented approach in a single pass from the dataset. In our proposed system, we use M-HDT for indexing the nodes and edge labels. Then, we employ gRePair algorithm for identifying the grammar from the RDF graph. Afterward, the system improves the performance of $k^{2}$-trees by introducing a more efficient algorithm to create the trees and serialize the RDF datasets. Our experiments affirm that the proposed gRDF scheme can substantially achieve at approximately 26.12%, 13.68%, 6.81%, 2.38%, and 12.76% better compression ratio when compared with the most prominent state-of-the-art schemes such as HDT, HDT++, $k^{2}$-trees, RDF-TR, and gRePair in the case of real-world datasets. Moreover, the processing efficiency of our proposed scheme also outperforms others.

## 1. Introduction

Nowadays, the RDF is a widely used standard data model for storing a large amount of semantic data. This RDF model can store and make connections among datasets from various sources such as scientific data, business knowledge, demographic information, social networks, and Wikipedia. For example, a DBPedia dataset not only contains Wikipedia data but also incorporates links to other datasets on the Web. Various applications can exploit the heterogeneous connection among datasets to gather additional knowledge. One of the most prominent ways to store the knowledge of the world is the knowledge graph (KG). It is used by a number of numerous applications such as dialog system [1,2], question answering [3,4], and search [5]. Therefore, the growth of KG is increasing, having millions of entities and their relationships. KG is represented by the RDF data model having a finite number of triples. A triple represents a relationship between a subject (S) and a object (O) via a predicate (P). However, managing and accessing the RDF data efficiently is not trivial, due to the enormous size of datasets, and raises several serious challenges. RDF data needs to be compact and comprehensive, saving communication bandwidth and storage, yet it should preserve the integrity of the data. Therefore, RDF compression has become an increasingly important research area due to the continuous expansion of structured RDF data. There are several approaches that are used to achieve lossless RDF compression. Some universal data compression schemes, such as bzip2 (http://www.bzip.org/, accessed on 23 March 2022) and LZMA (http://www.7-zip.org/, accessed on 23 March 2022), can be employed for the RDF data [6]. However, these approaches alter the structure of the RDF dataset and reduce the size of the data significantly. Some RDF compression approaches that alter the serialization of RDF data but preserve the structure of the data, such as HDT [7], HDT-TR [8], $k^{2}$-triples [9], and lean graphs [10], can be used for reducing the size of the RDF data. Another scheme, such as rule-based RDF compression considered as logical compression, is also introduced to reduce the triple number substantially from the RDF dataset [11,12]. Elimination of the redundancy in the ontology is also a logical compression scheme that interprets the semantics of OWL (http://www.w3.org/TR/owl-features/, accessed on 23 March 2022) (Web Ontology Language) [13]. Despite achieving the compressed RDF dataset by using the existing approaches, there are very few studies that focus on the structure of the graph in an RDF dataset. For example, the statistical redundancy of the RDF dataset is exploited in the case of a universal compression scheme, and the datasets are considered as an ordered character series. However, the RDF dataset is usually a graph, and the ordering of edges and nodes are extraneous for the semantics of the dataset, although there are very few studies that consider this sort of information and they constrict fixed simple graph structure of the dataset, which is less efficient, while compressing the RDF dataset. For example, [11] compresses the graph structures that are star-shaped and reoccur having changing center nodes with a single triple at an RDF dataset. This approach performs well when the dataset occupies many various nodes that are sharing the abundant same neighbors. However, this scheme cannot be used when such a structure is not present in the RDF dataset. On the other hand, RDF graphs are serialized according to the byte sequences, which produces syntactic redundancy. Existing techniques, such as RDF/XML [14], HDT [7], and HDT-TR [8], consider the syntactic redundancies of the graph structure in the RDF dataset. However, if the graph pattern knowledge is not present in the RDF dataset, these schemes cannot employ the common structure of the graph that is shared by the various instances of a single graph pattern. On the other hand, HDT encodes all the triples by using the tree-shaped triples; one per different subject by using bit and compact integer sequences. However, it does not consider the structural redundancies, i.e., common patterns when explaining the subjects, as the RDF data comes with various levels of structured and unstructured data. Moreover, many studies have proposed techniques for constructing a dictionary [15,16] to compress the RDF data that address the redundancy in URI properties. Some researchers exploit the semantics of an RDF dataset to perform syntax-based compression [17,18,19]. In addition to that, there are several approaches for processing the graph, ranging from tree-based [20] to context-free grammar [21] techniques. However, none of the above approaches focus enough to reduce the compression ratio, runtime, and redundancy of triple components. On the other hand, to the best of our knowledge, very little research work has been carried out previously to integrate the gRePair algorithm with the RDF compression algorithm for achieving better compression in the RDF dataset.

Therefore, in this study, we propose a novel approach named grammar-based RDF (gRDF) for efficiently compressing the RDF dataset by utilizing the gRePair [21] algorithm, which is one of the best graph compression schemes. This paper is an extension of our previous published work [22]. The key contributions of this paper are summarized as follows.

- We improve the performance of HDT by introducing modified HDT (M-HDT) to identify and hold predicates and graph patterns. It compresses the RDF dataset by considering the redundancy of the data. Therefore, our proposed scheme can optimize the use of memory space and reduce the loss of the data by employing a single-pass operation in the RDF dataset.
- We employ the gRePair algorithm, which is one of the best graph compression schemes, to the RDF dataset after indexing it by using our proposed M-HDT scheme in our proposed gRDF scheme.
- We store the remaining graph in the $k^{2}$-trees. In our proposed scheme, we develop an efficient algorithm for $k^{2}$-trees to achieve a more compressed RDF dataset with reduced run time.
- Extensive experiments were carried out to validate the performance in terms of compactness and processing efficiency.

The rest of the paper is organized as follows. Section 2 describes background information and explains the overview of the existing approaches. We represent the architecture of our proposed model and its associated features in Section 3. Then, Section 4 explains the details of the experimental settings and provides the results of our comprehensive evaluation. Finally, we conclude our investigation in Section 5 by summarizing our findings and including the suggestions for future research.

## 2. Related Work

The World Wide Web (W3C) introduced the first standard of RDF in 2004. From February 2014, we have been using the current version, RDF 1.1. It is employed to depict semantic information and link the data. A set of triples are used to represent the RDF data, where the subject is connected with the object via a predicate. We employ nodes to represent the subjects and objects and edges to denote the predicates in a set of triples for generating the graph structure of RDF. Therefore, the structure defined by the RDF is a natural representation of a graph similar to the relationship of XML with trees. The values for the subject, predicate, and object of an RDF graph are long strings, such as URIs. As RDF graphs compress the semantic information of the dataset, most of the existing approaches [6,23,24] use a dictionary to map the possible values into integer and illustrate the graph by using a set of triples represented in integer format. This leads to two different approaches for compressing the RDF dataset: syntactic compression and semantic compression. Syntactic compression uses a compressed string dictionary to identify and remove the symbolic redundancy from the RDF graph. HDT [7] is the pioneer scheme in this family that efficiently encodes the underlying graph to the header (description of the metadata in a dataset), dictionary (maps the value of the triple into ID), and triples (encode the RDF graph). The header is used for the processing and the discovery of the dataset. On the other hand, the dictionary employs a prefix tree to reduce the space for storing the URIs. This scheme at first groups the triple by the subject and then by using the predicate which is denoted by using the bit sets and the arrays of ID. Ref. [7] employs a predicate family that combines the frequently occurring predicates and extends HDT to HDT++ [18]. HDT++ stores the predicates’ family ID including the object ID instead of the predicate ID of each triple. It achieves better compression than HDT for the highly structured dataset. Another scheme, known as $k^{2}$-triple, uses a separate adjacency matrix for representing each predicate in the graph structure of the RDF dataset during RDF compression [9,23]. It employs $k^{2}$-trees to store the triples for handling the large RDF graphs by using an in-memory store of data. These techniques are considered the best RDF compressor in the current state-of-the-art RDF compression scheme [25]. Another RDF compression approach named RDF-TR (RDF-triples re-organizer) identifies and removes the structural regularities in an RDF dataset to underpin the nature, which is schema relaxed, of the RDF dataset [8]. It groups the subjects having the same predicates and recodes those predicate-related objects. In addition to that, it accommodates HDT and $k^{2}$-trees during the compression of RDF datasets. The RDFCSA [26] and OFR [27] are the most recent physical compressor. RDFCSA speeds up the data extraction process but it needs large space. Therefore, it does not perform better for RDF compression. Then, it uses compressed suffix array (CSA) to improve the performance [28]. Though it can compete with HDT in the case of effectiveness, it cannot achieve the compression ratio similar to $k^{2}$-triples. On the other hand, OFR mainly concentrates on reducing the storage space, while it does not consider the retrieval of triples. It isolates the triples into six sub-dictionaries, in which, at first, the dictionary is partitioned by subject, predicate, and object. Then, it creates the dictionaries to occupy each distinct class of triples. These dictionaries are delta- and run-length-compressed [29]. The triples are ordered by run-length and (O,S) values. They use delta compression to handle multiple objects. In the case of consecutive subjects, they employ non-decreasing order. In the second stage of compression, the triples and the dictionaries are again compressed. They use zip and 7zip to eliminate all the remained redundancy after employing the OFR scheme. Compression achieved by OFR incorporated with zip and 7zip outperforms HDT+zip and HDT+7zip. However, this achievement is not enough to compare whether a standalone OFR scheme can improve the performance of HDT.

On the other hand, semantic compression replaces the redundant parts of the graph for reducing the number of triples during compression. Ref. [21] proposes the gRePair algorithm, which is the extension of the RePair algorithm, to reduce the graph based on grammar. It creates the grammar according to the relationship in the graph and alters the original graph based on the rules of equivalent grammar by another graph. If there is a large number of redundancies in the object–predicate and subject–predicate pair relationships, and there are very few predicates in the graph, then the gRePair algorithm is more efficient. The performance of this algorithm has been compared with the $K^{2}$-trees method, and gRePair achieves 10 times better compression with respect to the $K^{2}$-trees method in a graph having one rdf:type predicate. To the best of our knowledge, no evaluation has been performed previously using a complete large real-world dataset with many predicates. However, [10,30] uses the concept of lean subgraph, which maps the redundant blank nodes into labels that are already present in the graph or other blank nodes. The rules, constraints, and queries are analyzed in [31] for minimizing the graph but do not include any practical results. Though [11] concludes that this scheme is not suitable for compressing the growing RDF dataset, on the contrary, [32] alters the redundant graph patterns by triples having newly constructed predicates and grammar that consists of rules which will be used for decompression. The rule-based compression method proposed by [11] employs the mining scheme to detect the frequent patterns that are then employed similar to the generative rules for deleting all the triples. However, there is no significant improvement in compression ratio by itself, and this compressor must be accompanied by HDT to achieve better performance. Furthermore, for capturing more semantic association in the RDF dataset, [12,33] employs more expressive rules to mine frequent patterns based on Horn rules. This compression scheme outperforms [11] in terms of compression ratio. Another rule-based compression scheme proposed by [34] employs OWL2RL for removing the redundant triples. To detect redundant subgraph patterns, it analyzes the entities of the subject–object. The compression ratio is not provided in this manuscript but they mentioned that their scheme identifies approximately 32.77% redundant triples.

## 3. Materials and Methods

**Figure 1:**
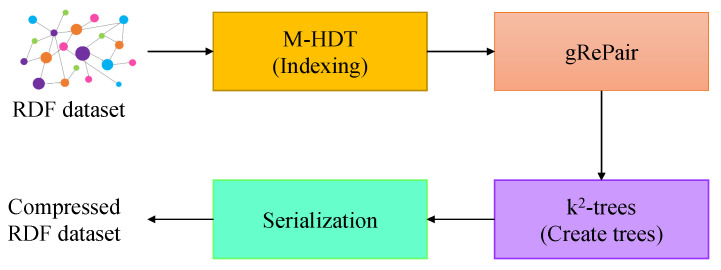
The proposed system architecture.

We employ gRePair algorithm for compressing the RDF dataset after indexing it by using our proposed modified HDT scheme in our proposed gRDF scheme. There are four steps in our system which are shown in Figure 1. In the first step, our gRDF scheme loads the RDF dataset into the memory. Then, the system indexes all the nodes and edges by using modified HDT (M-HDT). In the second step, we employ gRePair algorithm to generate grammar from the indexed RDF dataset. We build the *k*^2^-trees after employing the gRePair algorithm in our proposed system. In the final step, the system will serialize the graph in a sequence of *k*^2^-trees. Our system serializes each tree by using the edge labels ID. These steps are explained in more detail in the following.

### 3.1. M-HDT

HDT modularizes the data and utilizes the skewed nature of big RDF graphs [35,36] to reduce redundancy and accomplish large space savings. It has three main components; header, dictionary, and triple. Header stores the metadata of the RDF dataset and acts as an entry point of the datasets’ information to process and retrieve the conferred RDF graph according to the machine-readable and processable format. The dictionary arranges all the RDF terms (URI, blank nodes, and literals) into a catalog for providing a high level of compression in the RDF graph. There are three subsets of the elements (subject, *S*, predicate, *P*, object, *O*) in the RDF graph, represented as follows. Common subject–object is depicted as the set SO and mapped to [1,|SO|], non-common subject is denoted as S−SO and mapped to [|SO|+1,|S|], the non-common object is represented as O−SO and mapped to [|SO|+1,O], and predicate is denoted as *P* and mapped to [1,|P|]. However, the triple encodes the RDF graph compactly into a set of triples and reduces the noise due to the repetitions and long labels. It encodes the RDF triples into three IDs for the corresponding subject, predicate, and object terms represented in the dictionary.

**Figure 2:**
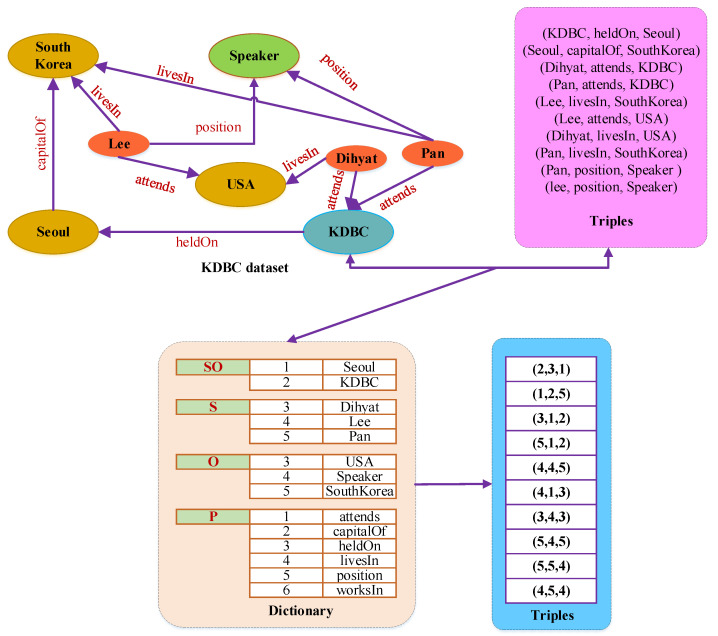
Dictonary and triple components of HDT for KDBC dataset.

Figure 2 shows an example of the dictionary and triples of HDT for the KDBC dataset. In this figure, the dictionary is created based on the RDF graph of the KDBC dataset and its associated triples. After that, the triples are encoded according to the triple components defined in the dictionary. Therefore, the HDT dictionary supports faster lookup and there is no possibility of ambiguity. However, there are some possibilities of redundancy such as structural regularities in the RDF graph of the dataset. In this study, we have modified the HDT to discover the frequent pattern by using our proposed M-HDT structure-oriented approach.

**Figure 3:**
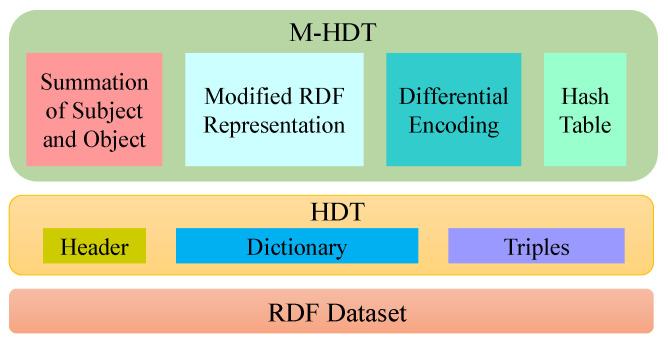
The architecture of the proposed modified M-HDT.

We use vector addition and subtraction for defining the operation of differential encoding in M-HDT. We employ this operation for two graphs to reduce the transmitted elements during compression and extract the hidden elements during decompression. We have used negative differential operation during compression and positive differential operation during decompression. Both negative and positive differential operation need two input vectors, $X=(x_{1},\;\ldots ,\;x_{i},\;\ldots ,\;x_{n})$ and $Y=(y_{1},\;\ldots ,\;y_{i},\;\ldots ,\;y_{n})$, where $x_{i}$ and $y_{i}$ represent the subject and object, respectively. These two vectors then return vector *Z*. Figure 3 represents the architecture of the M-HDT for detecting the frequent pattern to reduce the structural similarities.

- **Negative differential operation.** It keeps the elements of *A* by replacing the elements that are similar to *B*. These similar elements are represented by the empty string by using the following equation:
  (1) $$Z=A-B=\left(z_{1},\ldots ,z_{i},\ldots ,z_{n}\right)$$
  where
  $$\Gamma_{i}\in \left[1,n\right],z_{i}=\left\{\begin{array}{cc} “\;” & \;\mathrm{if}\;\left(A_{i}=B_{i}\right)\\ A_{i} & \;\mathrm{else}\; \end{array}\right.$$
  Thus, if A=(1,5,8) and B=(3,5,9) then Z=A−B=(1,“”,8).
- **Positive differential operation.** It keeps the elements of *A* that are not empty. In other cases, it returns the elements having similar indexes to *A* from *B*. The mathematical representation of the positive differential operation is depicted as follows:
  (2) $$Z=A+B=\left(Z_{1},\ldots ,Z_{i},\ldots ,Z_{n}\right)$$
  where
  $$\Gamma_{i}\in \left[1,n\right],Z_{i}=\left\{\begin{array}{cc} “\;” & \;\mathrm{if}\;\left(A_{i}\neq “\;”\;\;\;\right)\\ A_{i} & \;\mathrm{else}\; \end{array}\right.$$
  For example, A=(5,“”,“”) and B=(4,2,3) then Z=(A+B)=(5,2,3).

We use the hash table to represent the differential encoding for detecting a frequent pattern in M-HDT. It stores the list of subjects and objects of each graph where the summation of subject and object value (SSOV) is the key. For each subject of the triple, we create the SSOV by adding the subject and object. This value replaces the predicates and returns the graph structure in a compressed format named modified RDF representation (MRR), depicted as $<SSOV,\{subject,\;object_{1},\;object_{2},\;\ldots \}>$. After generating the MRR, the system finds whether the pattern has already been present in the other graph or not. A similar graph pattern can be identified by using the following definition. Let $G=g_{1},\;g_{2},\;\ldots ,\;g_{n}$ be the set of graphs in the KDBC dataset, $G_{p}=\left\{p_{i}\in P|i\leq n\right\}$ be a subset of *P*, and $G_{i}\in KDBC$ be the graph in the dataset. Thus, $G_{p}$ is a graph pattern of $G_{i}$ if and only if

(3) $$\exists p_{i}\left(\in G_{p}\to p_{i}\in G_{i}\right)$$

**Figure 4:**
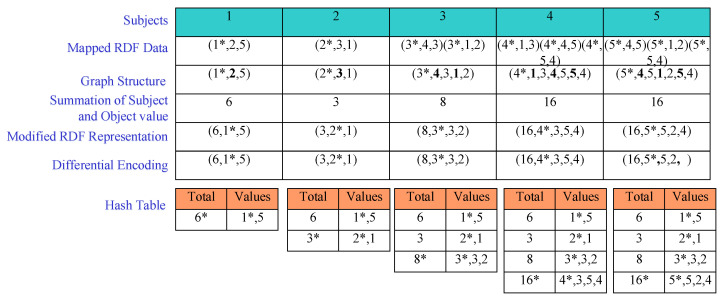
An example of the M-HDT.

Here, *n* is the number of nodes in a graph. According to the $G_{p}$ graph pattern, each $G_{i}$ RDF graph is generated. Therefore, if there exists a similar graph pattern previously according to Equation (3), we employ the differential encoding operation. Thus, the system becomes lighter by altering all the redundant values into empty strings and the hash table is updated. Then, we construct the compressed RDF graph. The algorithm of M-HDT is depicted in Algorithm 1. For better understanding, we employ our proposed M-HDT scheme in the KDBC dataset, which is shown in Figure 4.
**Algorithm 1:** M-HDT algorithm **Input**: RDFTriple(T) **Output**: CompressedHashTable(CHT)   1: CHT (graphPattern(GP), Objectlist)   2: **foreach** Graph ∈ T   3:     GP. construct   4:     GraphN ← GBV + Graph. subject + Graph. objects   5:        **if** GP ∈ CHT **then**   6:            PreviuosGraph ← CHT. get (GP)   7:            CHT. put (GP, GraphN)   8:            GraphN ← GraphN-PreviousGraph   9:        **else**   10:            HT. put (GP, GraphN)   11:      **end if**   12: **return** GraphN

In Figure 4, at first the subjects are placed. Then, we store the mapped RDF data where the subjects of all the triples are represented by the $Subject^{*}$. The representation format of the mapped RDF data is $<(Subject^{*},\;Predicate,\;Object),\;\ldots >$. In the graph structure, the predicates are depicted in bold letters. Then, SSOV is employed. After that, the MRR is used to encode the RDF graph. For example, the triples having subject 4 can be represented as $\left(16,\;4^{*},\;3,\;5,\;4\right)$ by replacing the triples $\left(\left(4^{*},\;1,\;3\right),\left(4^{*},\;4,\;5\right),\left(4^{*},\;5,\;4\right)\right)$ following the MRR. Finally, the differential encoding operation is employed that uses positive or negative differential operation. For example, our proposed system reduces the triples having subject 5 to $\left(16,\;5^{*},\;5,\;2,\;\;\right)$ by applying negative differential operation. It removes object 4 from the triples having subject 5 as it is already present in the triples having subject 4 and they both have the same SSOV (16). The evaluation of the differential encoding operation is depicted in the hash table shown in Figure 4.

### 3.2. gRePair

**Figure 5:**
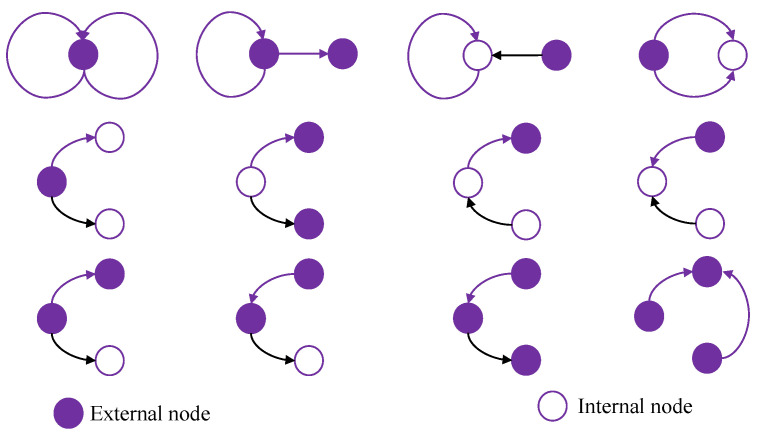
Different shapes of the diagram.

An RDF graph is a directed graph that can be represented by using a tuple, $RDF_{G}=\left(N,E,\gamma \right)$ where $N=\{n_{1},\;n_{2},\;\ldots ,\;n_{m}\}$ represents the set of all nodes, *E* denotes the edges, and γ:E→P depicts the mapping of the edge label. Our system uses gRePair algorithm proposed by [21] to create grammar, $G_{r}$, from the indexed $RDF_{G}$. We can define grammar as $G_{r}=\left(D_{s},\;D_{a}\right)$ where $D_{s}$ denotes the start graph which extends the mapping of the edge label, $\gamma^{\prime }:E\to P\cup D_{a}$ and $D_{a}$ defines the diagram sets that are employed to compress the RDF graph. On the other hand, a diagram is depicted as $d_{a}=\left(a_{i},\;a_{j}\right)$, where $a_{i}$ and $a_{j}$ represent two edge nodes that share at least one node. Therefore, each diagram is connected with three nodes. The nodes can be internal or external. An internal node is any node of a tree having child nodes. However, a node is called the external node if it occupies at least one edge node which does not exist in that diagram. We have used 33 various shapes of the diagrams in our system. Some examples are given in Figure 5.

**Figure 6:**
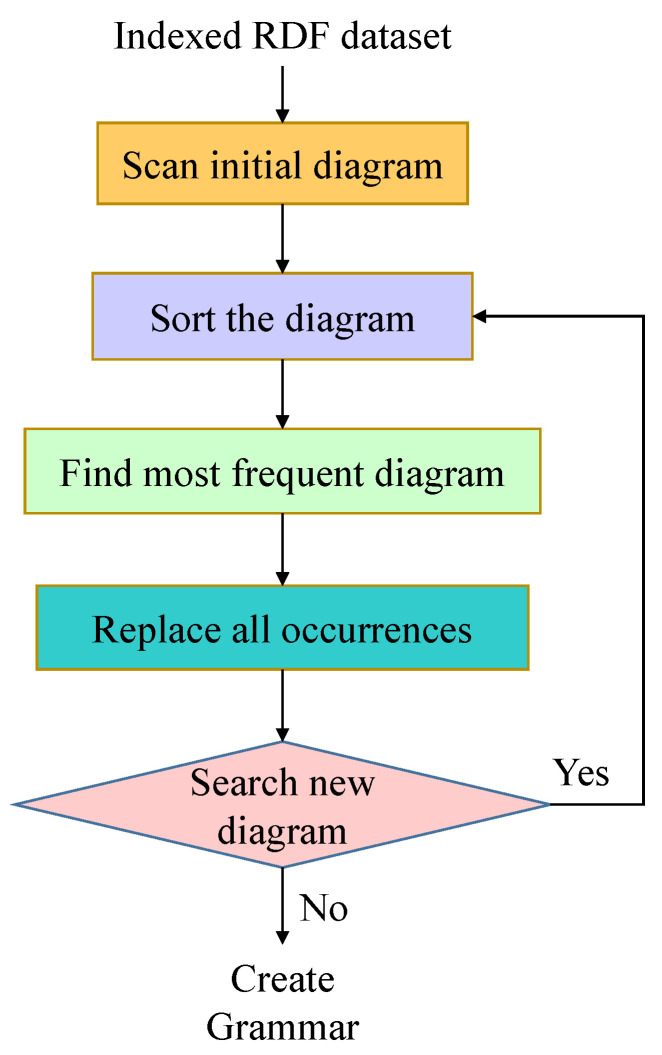
Steps of gRePair algorithm.

**Figure 7:**
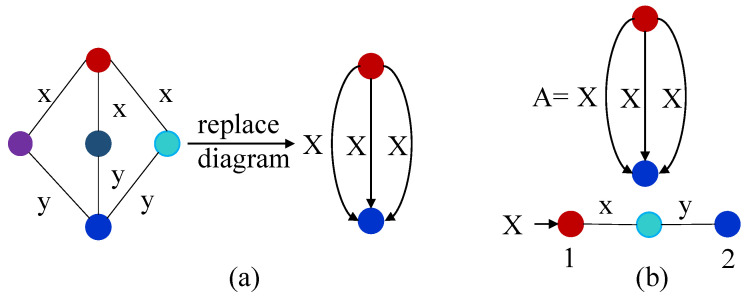
Example of gRePair algorithm. (**a**) Diagram replacement and (**b**) Grammar after the replacement of the diagram.

Our system uses the following steps shown in Figure 6 to employ the gRePair algorithm. For employing the gRePair algorithm, our proposed gRDF scheme at first iterates all the vertices of the RDF dataset. A diagram is considered a potential diagram if all the edge pairs are connected to a vertex. In the second step of the gRePair algorithm, our system sorts the diagram which appears at least twice in a descending order based on the priority queue. In the third step, the system will try to determine the most frequent diagram and remove it from the queue. The frequent diagrams within the graph are replaced by the non-terminal edges which are later added to the list for further serialization in the fourth step. As new edges are introduced, a new diagram may also generate. The vertices are searched to identify the new diagram only when it is connected with at least one newly introduced non-terminal edge. If a new diagram is identified, it will insert into the queue and repeat the process from step two as the queue is not empty. The algorithm of gRePair for the replacement of the occurrences is shown in Algorithm 2. An example of gRePair algorithm is represented in Figure 7.
**Algorithm 2:** Replacement of the occurrences in the gRePair algorithm **Input**: $RDF_{G}=\left(N,E,\gamma \right)$ **Output**: $G_{r}$   1: $I(d_{a})\leftarrow$ Index of all the occurrences that are not overlapped for each diagram, $d_{a}$ in $RDF_{G}$   2: **while**
$\left|I(d_{a})>1\right|$
**do**   3:      Select a diagram that is most frequent, $M_{f}$   4:      Replace each occurrence at $I(M_{f})$ by a new edge in $D_{s}$   5:      Occurrence list update   6: **end while**   7: **return**
$G_{r}$

In this example, we considered one edge-labeled graph at the left side of Figure 7a, in which we will identify a diagram having at least one common node. There are three occurrences of the diagram in that graph which have one *x* and *y* edge. Moreover, it also has another three occurrences which have two *x* and *y* edges, but these occurrences are overlapped. However, in that diagram, there should be, at most, one occurrence that is not overlapped. Therefore, we use a non-terminal edge label as *X* to alter every occurrence of the x/y diagram which is shown at the right side of Figure 7a. The grammar of this graph is represented in Figure 7b.

### 3.3. $k^{2}$-Trees

**Figure 8:**
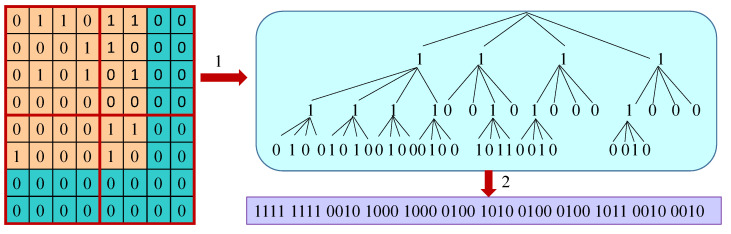
An example of the matrix encoding of a $k^{2}$-tree (1) and its serialization (2).

A start graph, $D_{s}$, and the set of all diagrams, $D_{diagram}$, are created from the grammar produced by the gRePair algorithm. In $S_{start}$, for each label of edge, our system creates an adjacency matrix according to [21]. The dimension of the matrix is |N|×|N| and the cell of the matrix is the edges between the row (subject) and column (object) indexes. If there is an edge label between the subject and the object that exists, then our system assigns 1 for denoting that cell. The generated matrix is generally sparse. Our system generates the $k^{2}$-trees [23] from these matrices. $k^{2}$-trees compress the dictionary similar to the HDT scheme. They partitions the predicates of RDF datasets vertically and create the disjoint subsets for the pair of subject–object. After that, these subsets are compressed highly because they use binary matrices (i.e., if the corresponding triple presents in the RDF dataset, it is marked by 1). Figure 8 denotes the resulting $k^{2}$-trees for an RDF graph and its serialization. It is an 8×8 matrix whose two right-most columns and two bottom-most rows are occupied by zeroes for reaching the needed matrix sizes, though they are not used to encode the current vertex. From the matrix, the conceptual tree and its serialization are created, and are located in the right side and the bottom of the tree (Figure 8). Before merging with other paths of the matrix, each path from the root to the leaves is created individually.

In our proposed system, we have modified the traditional $k^{2}$-trees algorithm and employed a more efficient algorithm during recursion. Our proposed algorithm at first iterates all the cells which have 1 value in a matrix. The path in $k^{2}$-trees is created for each of these iterated cells according to Algorithm 3. At first, the system resizes the matrix into $2^{m}\times 2^{m}$ where $2^{m}\geq \left|N\right|$. We use zero to fill the included rows and columns. If the dimension of a matrix is $2^{m}\times 2^{m}$, then this matrix can be divided into four equal-sized ($2^{m-1}\times 2^{m-1}$) sub-matrices, which is called a quadrant. These quadrants are denoted as follows:

- First quadrant: (0,0) to $\left(2^{m-1}-1,2^{m-1}-1\right)$.
- Second quadrant: $\left(0,2^{m-1}\right)$ to $\left(2^{m-1}-1,2^{m}\right)$.
- Third quadrant: $\left(2^{m-1},0\right)$ to $\left(2^{m},2^{m-1}-1\right)$.
- Fourth quadrant: $\left(2^{m-1},2^{m-1}\right)$ to $\left(2^{m},2^{m}\right)$.

The algorithm identifies those cells’ quadrant and includes a child node to the path before decreasing the size of the quadrant in a complete matrix. After that, our proposed Algorithm 3 is used to merge all the created paths. Then, the map denotes the $k^{2}$-trees that will be optimized during serialization.
**Algorithm 3:** Path creation and merging algorithm of $k^{2}$-trees **Input**: Matrix *N*, int *m*, *k* **Output**: list of paths   1: a1 = 0, b1 = 0, a2 = $2^{m}$, b2 = $2^{m}$   2: root = new $Tree_{Node}$()   3: presentNode = root   4: **for** Point x: N.getPoints() **do**   5:      Q = getQuadrant(x, a1, b1, a2, b2)   6:      C = new $Tree_{Node}$()   7:      presentNode.set(Q,C)   8:      presentNode = C   9:      shrinkBoundaries(a1, b1, a2, b2, Q)   10: Map(int,$Tree_{node}$) = map   11: **if** m == k OR $Tree_{node}$ == null **then**   12:      **return**   13: **for** C:$Tree_{node}$.getC() **do**   14:      map.get(k).add(C)   15: **for** C:$Tree_{node}$.getC() **do**   16:      merge(C, map, *k* + 1, *m*)   17: **return** map

### 3.4. Serialization

Our system serializes the start graph and diagram to serialize the grammar as follows.

**Figure 9:**
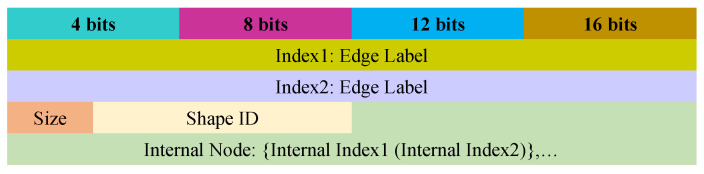
Serialization of the diagram where each row occupies 16 bits.

- **Start graph.** Our system serializes the start graph according to the sequence of $k^{2}$-trees. Every tree is preceded by its edge label ID (4 bytes). One bit is used to represent each tree node. Therefore, the system serializes the tree from the root to the leaf according to the sequence of bits denoted by its nodes. If there is an uneven number of nodes present in the tree, the system uses zero to pad the last byte. An example of serialization is shown in Figure 8, where we can use only 6 bytes to store the whole tree.
- **Diagram.** Our system serializes the diagram to reduce the size of the graph according to Figure 9. It consists of two indexes of edge labels. The IDs of the edge label denote the diagrams or properties ID that correspond to that edge label. For employing in the single internal node IDs and decoding the bytes number, our system uses two bits for the size flag. However, the diagrams shape ID is stored into the shape ID which consists of 6 bits. The diagrams shape can be one of the shapes among 33 different shapes. Moreover, the IDs of the internal nodes that occur in the diagram are stored in the internal node of Figure 9. The diagrams’ occurrence IDs are sorted according to the external node IDs. In addition to that, the mapping of the individual occurrences of the diagram and the internal nodes are implicitly sorted without occupying further space.

### 3.5. Decompression

During decompression, our system at first loads the dictionary. Then, the RDF triple is generated after loading all the $k^{2}$-trees of every terminal edge. After that, all the diagrams are iterated reversely. The non-terminal edges are ordered according to their connected vertices IDs. A single non-terminal is altered by the internal nodes and two edges according to this order. The internal nodes are interpreted in the accurate order as the non-terminal edges are ordered similar to the serialization. The created terminal edges are immediately transformed into the RDF graph according to the two edge labels contained in a diagram. The generated non-terminals are included in their diagrams list.

## 4. Discussion

We evaluated our proposed system performance by employing the prototype in Java on Linux OS (Ubuntu 20.04.3) consisting of Intel core i5-4690, 3.50 GHz, and 64 GB RAM. We used LUBM benchmark datasets (http://swat.cse.lehigh.edu/projects/lubm/, accessed on 23 March 2022) and two real-world publicly available datasets named ArchivesHub (http://data.archiveshub.ac.uk/, accessed on 23 March 2022) and ScholarlyData (https://old.datahub.io/dataset/scholarlydata/, accessed on 23 March 2022) to analyze the performance of our proposed scheme. For LUBM 1 and LUBM 10 datasets, the number of files we employed were 15 and 189, and for ArchivesHub and ScholarlyData datasets, the number of files we employed were 250 and 283 during the experiment.

For evaluating the performance of our proposed scheme, we measured the compression ratio, compression time, decompression time, and dictionary size by using HDT, HDT++, $k^{2}$-trees, HDT-TR, gRePair, and our proposed gRDF schemes. Our experiments considered heterogeneous linked open RDF datasets of various sizes. Table 1 shows the statistical description of the datasets that we used during our experimental analysis. The Lehigh University Benchmark (LUBM) dataset features the ontology of the university domain where all the entities of a university, such as students, professors, and courses, are depicted in the triple format. On the other hand, the ArchivesHub dataset consists of the archived data of UK institutions, and ScholarlyData comprises the dataset of the semantic web community for people, papers, organizations, and events concerned with the academic conferences.

**Figure 10:**
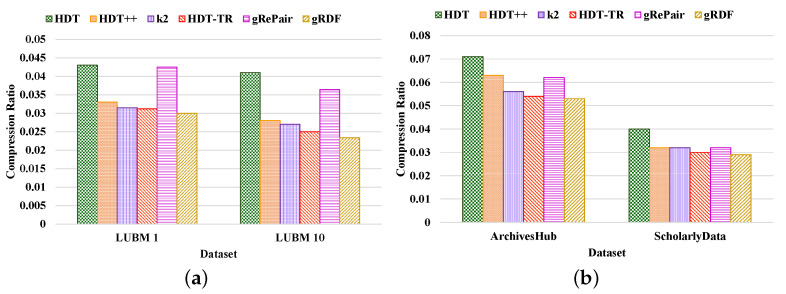
Compression ratio measured by various compression schemes of (**a**) LUBM and (**b**) real-world datasets.

**Figure 11:**
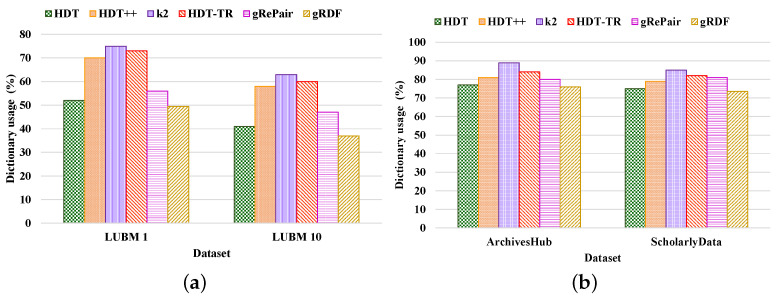
Analysis of dictionary size measured by various compression schemes of (**a**) LUBM and (**b**) real-world datasets.

**Figure 12:**
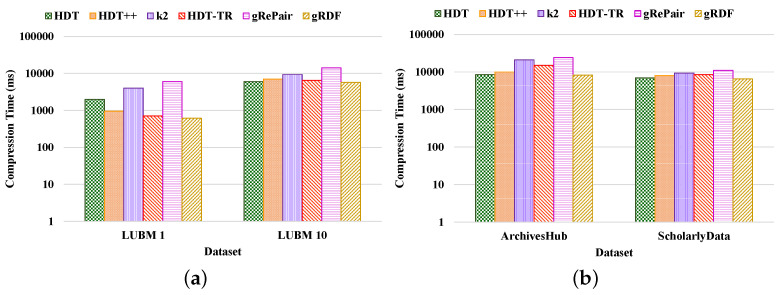
Compression time in logarithmic scale measured by various compression schemes of (**a**) LUBM and (**b**) real-world datasets.

**Figure 13:**
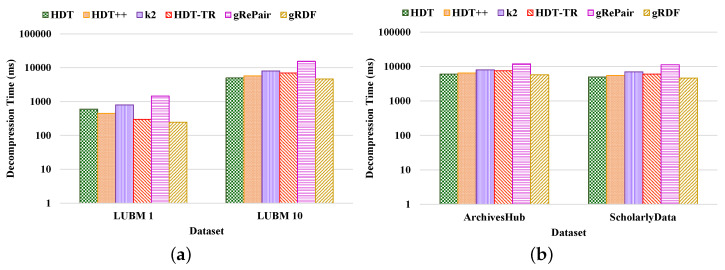
Decompression time in logarithmic scale measured by various compression schemes of (**a**) LUBM and (**b**) real-world datasets.

- **Compactness Results.** We compared the performance of our proposed system with the existing HDT [7], HDT++ [18], $k^{2}$-trees [9], RDF-TR [8], and gRePair [21] techniques for compact RDF serialization to analyze the efficiency of our proposed system. Figure 10 shows the compression ratio for different datasets compressed by the existing state-of-the-art techniques and our proposed system. The compression ratio measures the ratio of the number of triples that remain in the dataset after compression to the total number of triples. Therefore, a lower compression ratio indicates better performance. The figure proves that our proposed system achieves a better compression ratio than the other techniques. For example, our proposed system has achieved approximately 36.42%, 12.45%, 8.71%, 4.98%, and 32.31%; and 26.12%, 13.68%, 6.81%, 2.38%, and 12.76% better compression ratio than the existing HDT, HDT++, $k^{2}$-trees, RDF-TR, and gRePair schemes in the case of LUBM datasets and real-world datasets, respectively (Figure 10a). On the other hand, our system has approximately 30.23%, 9.09%, 4.76%, 3.84%, and 29.41% better compression ratio when using LUBM 1 dataset and 42.92%, 16.42%, 13.33%, 6.4%, and 35.71% better compression ratio when using LUBM 10 dataset than the existing HDT, HDT++, $k^{2}$-trees, RDF-TR, and gRePair schemes (Figure 10a). In addition to that, our system also has approximately 25.35%, 15.87%, 5.35%, 1.85%, and 14.51% better compression ratio when using ArchivesHub dataset and 27.5%, 9.37%, 8.98%, 3.33%, and 9.12% better compression ratio when using ScholarlyData dataset than the existing HDT, HDT++, $k^{2}$-trees, RDF-TR, and gRePair schemes (Figure 10b). This is because our proposed system can discover and remove the structural redundancies of the datasets before compressing the dataset efficiently. In addition to that, the hash table in the M-HDT can detect, hold predicates, and graph patterns to optimize the memory space usages. On the other hand, we have received the same dataset size after decompression by using our proposed scheme; this means that our proposed gRDF scheme does not lose any data during compression. Furthermore, we have also analyzed the percentage of gain in the compression ratios provided by our proposed gRDF scheme with respect to the best performing exiting scheme for various datasets [37]. From Figure 10, we have come to know that the RDF-TR scheme performs better among the existing schemes for all the experimented datasets. Therefore, our proposed gRDF scheme outperforms RDF-TR. After analyzing Figure 10a, we can conclude that for LUBM 1 and LUBM 10 datasets, our proposed scheme has achieved 3.84% and 6.4% gain. On the other hand, our proposed gRDF scheme has achieved 1.85% and 3.33% gain for ArchivesHub and ScholarlyData datasets, which is evaluated from Figure 10b.
  Then, we measured the space required to store the compressed dictionary in terms of the total size of the compressed dataset, which is shown in Figure 11. The dictionary replaces long terms of the RDF triples to the short IDs along with their references. It enormously compresses the RDF datasets as well as elevates the issues of scalability. From this figure, we can observe that most of the space used by the compared state-of-the-art techniques and our proposed scheme is consumed by the dictionary. The less the space used by the dictionary leads, the better the compression ratio. From this figure, we can observe that our proposed system can use the dictionary much better than others. For example, the average size of the dictionary of our proposed scheme in terms of all the real-world datasets is at approximately 75%, which is much better than other schemes (Figure 11b). Moreover, the average dictionary size of our system is approximately 1.29%, 6.17%, 14.60%, 9.52%, and 5.00%; 2.00%, 6.96%, 13.52%, 10.36%, and 9.25% better than the existing HDT, HDT++, $k^{2}$-trees, RDF-TR, and gRePair schemes in terms of ArchivesHub and ScholarlyData dataset (Figure 11b). In addition to that, the average dictionary size of our proposed system is approximately 4.80%, 29.28%, 34.00%, 32.19%, and 11.60%; 9.75%, 36.20%, 41.26%, 38.33%, and 21.27% better than the existing HDT, HDT++, $k^{2}$-trees, RDF-TR, and gRePair schemes in terms of LUBM 1 and LUBM 10 dataset (Figure 11a). This is because our proposed M-HDT can store the hash table in a compressed form by removing the frequent patterns of the RDF dataset. Moreover, the triples in the RDF can be represented in the star pattern and trees which are compressed well by using gRePair because during the replacement of the occurrences, it reduces the edge number in half. This saves processing resources and enables larger size dictionaries to be managed in a fixed main memory. However, we have also analyzed the percentage of gain in the dictionary size achieved by the proposed gRDF scheme in terms of best performing existing scheme among various datasets. Therefore, after analyzing Figure 11, we have come to know that the dictionary of HDT uses much less space among other existing schemes. However, the gain of our proposed scheme is 4.80% and 9.75% for LUBM 1 and LUBM 10; and 1.29% and 2% for ArchivesHub and ScholarlyData datasets (Figure 11a,b).
- **Processing efficiency.** We have analyzed the compression and decompression time of our proposed scheme in terms of the various datasets and compared the performance with respect to the state-of-the-art techniques. From Figure 12 and Figure 13, we can observe that our proposed gRDF scheme has better compression and decompression time than the other state-of-the-art schemes. This is because of the better dealing capacity for the redundant structure of the dataset as well as the use of simple data structure for identifying and building graph patterns. Moreover, gRePair has the capability to execute the finite automata without prior decompression in one pass by using a speed-up algorithm [21]. For example, our proposed gRDF scheme compressed the real-world datasets at approximately 4.51%, 17.77%, 51.31%, 37.02%, and 57.83% times faster than the existing HDT, HDT++, $k^{2}$-trees, RDF-TR, and gRePair schemes, respectively (Figure 12b). Moreover, it decompressed the real-world datasets at approximately 5.68%, 13.54%, 30.83%, 23.14%, and 55.28% times faster than the existing HDT, HDT++, $k^{2}$-trees, RDF-TR, and gRePair schemes, respectively (Figure 13b). On the other hand, after observing Figure 12 and Figure 13, we can observe that the difference between the compression and decompression time of our proposed scheme is larger than the other existing schemes when using the LUBM 1 dataset. In future work, we will try to resolve this issue.

## 5. Conclusions

The exponential growth of semantic web contents and the flexible paradigm of RDF demands compression to efficiently manage and reduce the size of RDF datasets. There are several approaches for compressing the RDF datasets; however, most of the existing approaches do not perform well in all dimensions, such as better compression ratio and runtime. In this study, we introduce a novel gRDF scheme for compressing the RDF dataset more efficiently with better runtime. We introduce M-HDT to index all the edges and node labels within the graph, which improves the performance of HDT by identifying the regularities and redundancies of the RDF datasets while taking the benefit of datasets structure where the same types of subjects are denoted via a similar set of properties, and the range of their values are little overlapped. We employ one of the most effective grammar-based graph compression gRePair algorithms to create grammar from the indexed RDF datasets. Moreover, we improve the performance of the traditional $k^{2}$-trees by introducing a more efficient algorithm for path creation and merging to create the trees. Our system then serializes each tree by using the edge labels ID. We use one bit to depict the node of the tree. Therefore, our system serializes the nodes from top to bottom in a sequence of bits. The performance of our proposed system has been evaluated on real-world datasets and compared with HDT and other state-of-art schemes. Simulation results affirm that our proposed system outperforms the other existing schemes for compactness as well as processing efficiencies. There are some limitations of our proposed gRDF scheme. For example, from the experimental analysis of the dictionary size for various datasets, we have come to know that the average size of the dictionary for the LUBM and the real-world datasets are approximately more than 37% and 75%. Therefore, if we reduce the size of the dictionary, we can achieve better compression. On the other hand, in this research, the query execution on the compressed graph is out of scope. In the future, we are planning to use embedding techniques during compression, because the embedding technique will represent the nodes and edges of the RDF graph in low-dimensional vector while maintaining the semantic properties and topological features. Therefore, the obtained vector will be task-independent so that it can be reused for other applications. Moreover, we will also focus on overcoming the limitation of our proposed scheme.

## Figures and Tables

**Table 1:** Dataset statistics.

Name	Triples	Resources	Classes
LUBM 1	100545	17209	15
LUBM 10	1272577	207461	15
ArchivesHub	1361815	135643	46
ScholarlyData	859840	95016	46

## Data Availability

Not applicable.

## References

[1] He H., Balakrishnan A., Eric M., Liang P. (2017). Learning symmetric collaborative dialogue agents with dynamic knowledge graph embeddings. arXiv.

[2] Young T., Cambria E., Chaturvedi I., Zhou H., Biswas S., Huang M. Augmenting end-to-end dialogue systems with commonsense knowledge. Proceedings of the AAAI Conference on Artificial Intelligence.

[3] Berant J., Chou A., Frostig R., Liang P. Semantic parsing on freebase from question-answer pairs. Proceedings of the 2013 Conference on Empirical Methods in Natural Language Processing.

[4] Lopez V., Unger C., Cimiano P., Motta E. (2013). Evaluating question answering over linked data. J. Web Semant..

[5] Singhal A. (2012). Introducing the Knowledge Graph: Things, Not Strings. Official Google Blog.

[6] Fernández J.D., Gutierrez C., Martínez-Prieto M.A. RDF compression: Basic approaches. Proceedings of the 19th International Conference on World Wide Web.

[7] Fernández J.D., Martínez-Prieto M.A., Gutierrez C. (2010). Compact representation of large RDF data sets for publishing and exchange. The Semantic Web—ISWC 2010, Proceedings of the International Semantic Web Conference, Shanghai, China, 7–11 November 2010.

[8] Hernández-Illera A., Martínez-Prieto M.A., Fernández J.D. (2020). RDF-TR: Exploiting structural redundancies to boost RDF compression. Inf. Sci..

[9] Álvarez-García S., Brisaboa N.R., Fernández J.D., Martínez-Prieto M.A. (2011). Compressed k2-triples for full-in-memory RDF engines. arXiv.

[10] Iannone L., Palmisano I., Redavid D. (2005). Optimizing RDF storage removing redundancies: An Algorithm. Innovations in Applied Artificial Intelligence, Proceedings of the International Conference on Industrial, Engineering and Other Applications of Applied Intelligent Systems, Bari, Italy, 22–24 June 2005.

[11] Joshi A.K., Hitzler P., Dong G. (2013). Logical linked data compression. The Semantic Web: Semantics and Big Data, Proceedings of the Extended Semantic Web Conference, Montpellier, France, 26–30 May 2013.

[12] Sultana T., Lee Y.K. Expressive Rule Pattern Based Compression with Ranking in Horn Rules on RDF Style KB. Proceedings of the 2021 IEEE International Conference on Big Data and Smart Computing (BigComp).

[13] Grimm S., Wissmann J. (2011). Elimination of redundancy in ontologies. The Semantic Web: Research and Applications, Proceedings of the Extended Semantic Web Conference, Crete, Greece, 29 May–2 June 2011.

[14] Beckett D., McBride B. (2004). RDF/XML syntax specification (revised). W3C Recomm..

[15] Yuan P., Liu P., Wu B., Jin H., Zhang W., Liu L. (2013). TripleBit: A fast and compact system for large scale RDF data. Proc. Vldb Endow..

[16] Martínez-Prieto M.A., Fernández J.D., Cánovas R. (2012). Querying RDF dictionaries in compressed space. ACM SIGAPP Appl. Comput. Rev..

[17] Fernández J.D., Martínez-Prieto M.A., Gutiérrez C., Polleres A., Arias M. (2013). Binary RDF representation for publication and exchange (HDT). J. Web Semant..

[18] Hernández-Illera A., Martínez-Prieto M.A., Fernández J.D. Serializing RDF in compressed space. Proceedings of the 2015 Data Compression Conference.

[19] Sultana T., Qudus U., Umair M., Kim T., Morshed M.G., Lee Y.K. Efficient Frequent Pattern Management and Compression System in Multiple Named Graphs. Proceedings of the KIISE Korea Computer Congress 2020 (KCC 2020).

[20] Brisaboa N.R., Ladra S., Navarro G. (2009). k^2^-trees for compact web graph representation. String Processing and Information Retrieval, Proceedings of the International Symposium on String Processing and Information Retrieval, Saariselkä, Finland, 25–27 August 2009.

[21] Maneth S., Peternek F. (2018). Grammar-based graph compression. Inf. Syst..

[22] Sultana T., Lee Y.K. Employing Graph Compression Technique for Efficiently Compressing RDF Knowledge Graphs. Proceedings of the Korean Database Conference 2021 (KDBC 2021).

[23] Álvarez-García S., Brisaboa N., Fernández J.D., Martínez-Prieto M.A., Navarro G. (2015). Compressed vertical partitioning for efficient RDF management. Knowl. Inf. Syst..

[24] Martínez-Prieto M.A., Fernández J.D., Cánovas R. Compression of RDF dictionaries. Proceedings of the 27th Annual ACM Symposium on Applied Computing.

[25] Brisaboa N.R., Ladra S., Navarro G. (2014). Compact representation of web graphs with extended functionality. Inf. Syst..

[26] Brisaboa N.R., Cerdeira-Pena A., Farina A., Navarro G. (2015). A compact RDF store using suffix arrays. String Processing and Information Retrieval, Proceedings of the International Symposium on String Processing and Information Retrieval, London, UK, 1–4 September 2015.

[27] Swacha J., Grabowski S. (2015). OFR: An Efficient Representation of RDF Datasets. International Symposium on Languages, Applications and Technologies.

[28] Sadakane K. (2003). New text indexing functionalities of the compressed suffix arrays. J. Algorithms.

[29] Salomon D. (2004). Data Compression: The complete Reference.

[30] Meier M. (2008). Towards rule-based minimization of RDF graphs under constraints. Web Reasoning and Rule Systems, Proceedings of the International Conference on Web Reasoning and Rule Systems, Karlsruhe, Germany, 31 October 31–1 November 2008.

[31] Pichler R., Polleres A., Skritek S., Woltran S. (2010). Redundancy elimination on RDF graphs in the presence of rules, constraints, and queries. Web Reasoning and Rule Systems, Proceedings of the International Conference on Web Reasoning and Rule Systems, Bressanone/Brixen, Italy, 22–24 September 2010.

[32] Pan J.Z., Pérez J.M.G., Ren Y., Wu H., Wang H., Zhu M. (2014). Graph pattern based RDF data compression. Semantic Technology, Proceedings of the Joint International Semantic Technology Conference, Chiang Mai, Thailand, 9–11 November 2014.

[33] Gayathri V., Kumar P.S. Horn-rule based compression technique for RDF data. Proceedings of the 30th Annual ACM Symposium on Applied Computing.

[34] Guang T., Gu J., Huang L. (2016). Detect redundant rdf data by rules. Database Systems for Advanced Applications, Proceedings of the International Conference on Database Systems for Advanced Applications, Dallas, TX, USA, 16–19 April 2016.

[35] Ding L., Finin T. (2006). Characterizing the semantic web on the web. The Semantic Web—ISWC 2006, Proceedings of the International Semantic Web Conference, Athens, GA, USA, 5–9 November 2006.

[36] Theoharis Y., Tzitzikas Y., Kotzinos D., Christophides V. (2008). On graph features of semantic web schemas. IEEE Trans. Knowl. Data Eng..

[37] Fernández N., Arias J., Sánchez L., Fuentes-Lorenzo D., Corcho Ó. (2014). RDSZ: An approach for lossless RDF stream compression. The Semantic Web: Trends and Challenges, Proceedings of the European Semantic Web Conference, Crete, Greece, 25–29 May 2014.

